# A Treatise on Poisons in Relation to Medical Jurisprudence, Physiology, and the Practice of Physic

**Published:** 1845-05

**Authors:** 


Art. VI.—*A Treatise on Poisons in relation to Medical Jurisprudence, Physiology, and the Practice of Physic.* By Robert Christison, M. D., F. R. S. E.; Professor of Materia Medica in the University of Edinburgh; Fellow of the Royal College of Physicians, &c.; Member of the American Philosophical Society, of the Royal Academy of Medicine of Paris, &c., &c., &c. First American, from the Fourth Edinburgh Edition. Philadelphia: Ed. Barrington & Geo. D. Haswell. 1845. 8vo. pp. 756.

The subscribers of the Select Medical Library and Bulletin of Medical Science are already in possession of this elaborate and admirable treatise, the editor, with his usual judgment, having selected it for the beginning of a new year. It is a work the want of which has long been felt in this country, and appearing now in an unexpensive form it may be expected in due season to take its place in the libraries of all reading physicians. No one need hesitate about its purchase on account of any apprehension that it may be superceded in any reasonable time by a more perfect work, since it would be difficult to point out in it any deficiency, or to show how in any particular it might be improved. For the student seeking to store his mind with all that relates to the subject of poisons, and equally for the practitioner who may have occasion to refresh his memory concerning the tests and antidotes of these agents, it is safe to say that it is one of the most finished and satisfactory guides that any language could furnish. Higher authority than that of Christison could not easily be found on any point of the profession towards which he has directed his mind, but having made poisons a matter of special study for many years, during which he has enjoyed ample opportunities for becoming familiar with all the details of the subject, a work abounding in original observations and opinions, exhibiting the science of toxicology in its present advanced state, and entitled, therefore, to take the first rank in its class, is what might have been looked for from his pen. We congratulate our readers on the opportunity now afforded them of procuring such a treatise.

The work is divided into two parts, the first treating of general poisoning, and the second of individual poisons. Under the first head the physiological action of poisons is discussed. This may be either local or remote. The local effects of poisons are various, some acting chemically and corroding the parts to which they are applied, others producing irritation or inflammation, and a few making a peculiar impression on the sentient extremities of the nerves, unattended by any manifest change of structure. The alkalis and concentrated mineral acids act in the manner first mentioned; alcohol, cantharides, and tartar emetic inflame or irritate the skin and other tissues to which they are directly applied; arsenic, euphorbium, and the diluted mineral acids affect the stomach in the same way. Of the third mode, by nervous impression, we have fewer unequivocal instances, but the impression of the aconitum napellus, or monkshood, upon the tongue and lips, when chewed, a sense of numbness and tingling, lasting for some hours, and unconnected with any affection of the general nervous system, is considered an example, as is also the numbness of the end of the finger from contact with strong hydrocyanic acid, a numbness as from the local action of intense cold. It is seldom that death results from the local action of a poison, whether corrosive, inflammatory, or nervous; but the effects are radiated to distant organs, the suspension of the function of which is the cause of the fatal termination. Their remote action, therefore, is far the most important.

We have an example, and a very striking one, of this species of action in oxalic acid. It is corrosive, and cases are recorded in which it has produced perforations in the stomach; yet it never kills by destroying the function of that organ, for in some instances no trace of injury in the stomach could be found when death had followed a large dose of the poison. This acid has been known to kill a man in ten minutes, and a dog in as short a time as three minutes, but in such instances it was considerably diluted, which circumstance favored the remote action of the poison while it diminished, in the same proportion, its local action. Let us look for a moment at the way in which the influence of a poison is conveyed from one organ to another.

It is plain that there are but two ways in which this can be accomplished; the poison must be absorbed, or must act through the medium of the nerves upon distant organs. In the infancy of toxicology all poisons were believed to act in the latter way; but at present the other doctrine is more prevalent, while a recent theory, combining both views, represents that, although many poisons do enter the blood, their operation nevertheless is directly upon the nerves of the blood-vessels, and thus remotely through them upon the brain or other organs.

In the case of poisons that manifestly injure the structure of the organ to which they are applied, the affection of remote organs is undoubtedly sympathetic. Such is the fact with regard to the mineral acids, in which dilution lessens and even takes away altogether their remote poisonous action. But it is by no means so clear that distant organs sympathise with the peculiar local impressions, styled nervous, which are unattended by any visible derangement of structure. It is in relation to this that the chief disputes among physiologists have arisen, and we agree with our author that the existence of this variety of action by sympathy is doubtful. At one time it was pretty generally received as the true explanation of the mode in which many poisons affected the system at large, and facts such as the following were thought to be inexplicable upon any other principle:

"Magendie, speaking of the pure hydrocyanic acid, compares it in point of swiftness of action to the cannon ball or thunderbolt. In the course of certain experiments made not long ago with the diluted acid by Dr. Freer, Mr. Macauly and others, to decide the true rapidity of this poison, several dogs were brought under its influence in ten, eight, five, and even three seconds; during an experimental inquiry I afterwards undertook for the same purpose, I remarked on one occasion that a rabbit was killed outright in four seconds; and Mr. Taylor has more recently stated, that he has seen the effects induced so quickly in cats, that there was no sensible interval of time between the application of the poison to the tongue and the first signs of poisoning. Strychnia, the active principle of nux-vomica, acts sometimes with a speed little inferior to that of hydrocyanic acid; for Pelletier and Caventou have seen its effects begin in fifteen seconds. Alcohol, according to Sir B. Brodie, also acts on animals with equal celerity; for when he intoduced it into the stomach of a rabbit, its effects began when the injection was hardly completed."

But, as remarked by Dr. Christison, the progress of physiological discovery has lately brought the soundness of these views into question.

"Some years ago Dr. Hering of Stuttgardt showed that the round of the circulation may be accomplished by the blood much more speedily than had been conceived before; for the ferrocyanide of potassium, injected into the jugular vein of a horse, was discovered by him throughout the venous system at large in the short space of twenty or thirty seconds, and consequently must have passed in that period throughout the whole double circle of the pulmonary and systemic circulation. This discovery at once shook the validity of many, though not all, of the facts which had been previously referred to the agency of nervous transmission on the ground of the celerity with which the effects of poisons are manifested. More recently an attempt has been made by Mr. Blake to prove, that the circulation is so rapid as to admit even of the swiftest cases of poisoning being referred to the agency of absorption. Mr. Blake, who is altogether opposed to the occurrence of nervous transmission in the instance of any poison, has found that ammonia, injected into the jugular vein of a dog, was indicated in its breath in four seconds; and that chloride of barium or nitrate of baryta, introduced into the same vessel, could be detected in the blood of the carotid artery in about sixteen seconds in the horse, in less than seven seconds in the dog, in six seconds in the fowl, and in four seconds in the rabbit. These interesting discoveries, however, will not absolutely destroy the conclusiveness of all the facts quoted above in support of the existence of a sympathetic action."

No such difficulties attend the other doctrine. The action of poisons through the medium of the circulation is established by conclusive evidence, a portion of which our author thus sums up:

"First, they disappear during life from the shut cavities or other situations into which they have been introduced; that is, they are absorbed. Several clear examples to this effect have been related by Dr. Coindet and myself in our paper on oxalic acid. In one experiment four ounces of a solution of oxalic acid were injected into the peritoneal sac of a cat, and killed it in fourteen minutes; yet, on opening the animal, although none of the fluid had escaped by the wound, we found scarcely a drachm remaining. In recent times Professor Orfila has proved that various poisons, such as arsenic, tartar-emetic, and acetate of lead, disappear in part or wholly from wounds into which they had been introduced. Next, many poisons act with unimpaired rapidity, when the nerves supplying the part to which they are applied have been previously divided, or even when the part is attached to the body by arteries and veins only. Dr. Monro, *Secundus*, proved this in regard to opium; and the same fact has been since extended by Sir B. Brodie and Professor Emmert to wourali, by Magendie to nux vomica, by Coullon to hydrocyanic acid, by

Charret to opium, and by Dr. Coindet and myself to diluted oxalic acid. Magendie's experiment was the most precise of all: for, besides the communication with the poisoned part being kept up by a vein and an artery only, these vessels were also severed and reconnected by two quills. Farther, many poisons will not act when they are applied to a part of which the circulation has been arrested, even although all its other connections with the body have been left entire. This has been shown distinctly by Emmert in regard to the hydrocyanic acid; which, when introduced into the hind-leg of an animal after the abdominal aorta has been tied, produces no effect till the ligature be removed, but then acts with rapidity. An experiment of a similar nature performed by Mr. Blake with the wourali poison yielded the same result. Again, many poisons act with a force proportional to the absorbing power of the texture with which they are placed in contact. This is the criterion which has been commonly resorted to for discovering whether a poison acts through the medium of the blood. It is applicable, however, only when the poison acts sensibly in small doses; for those which act but in large doses cannot be applied in the same space of time over equal surfaces of different textures. The difference in the absorbing power of the different tissues has been well ascertained in respect to a few of them only. The most rapid channel of absorption is by a wound, or by immediate injection into a vein; the surface of the serous membranes is a less rapid medium, and the mucous membrane of the alimentary canal is still less rapid. Now it is proved of many poisons that, when applied in similar circumstances to these several parts or tissues, their activity is proportional to the order now laid down. Lastly, it has been proved of nux-vomica, that if the extract be thrust into the paw of an animal after a ligature has been tightened round the leg so as to stop the venous, but not the arterial circulation of the limb, blood drawn from an orifice in a vein between the wound and the ligature, and transfused into the vein of another animal, will excite in the latter the usual effects of the poison, so as even to cause death; while, on the contrary, the animal from which the blood has been taken will not be affected at all, if a sufficient quantity be withdrawn before the removal of the ligature. These interesting facts, which are capable of important practical applications, were ascertained by M. Verniere."

Still a question remains as to the action of the poisons after they have gained admission into the blood. It is not yet perfectly clear that they are transmitted in substance to the organs affected by them. A different theoretical view has been taken of the facts by Dr. Addison and Mr. Morgan, and as it is ingenious we give it in the words of our author. Their theory is—

"That the action may really take place by the poison producing on the sentient extremities of the nerves of the inner membrane of the blood-vessels a peculiar impression which is conveyed through the nerves to the part ultimately affected. They have endeavored to found this theory upon evidence, that the poison is not carried beyond the venous system; or that, if conveyed farther, it is carried incidentally, and not for the purpose of impregnating the textures of the organ which suffers. The evidence they have brought forward on this head is chiefly the following. 1. Poisons which act on a particular organ at a distance do not act more quickly when introduced into the artery which supplies it, than when introduced into its vein, or even into the principal artery of a distant part of the body. 2. If a poison be introduced into a great vein with a provision for preventing its passage towards the heart, it will act with as great rapidity, as if no obstacle of the kind existed. Thus, if the jugular vein, secured by two temporary ligatures, be divided between them and reconnected by a tube containing wourali, the animal will not be affected more quickly on the removal of both ligatures, than on removing only the ligature farthest from the heart. 3. The arterial blood of a poisoned animal is incapable of affecting another animal. Thus, if the carotid artery and jugular vein of one dog be divided, and both ends of each reciprocally connected by tubes with the divided ends of the corresponding vessels of another dog, and extract of nux-vomica be introduced into a wound in the face of one of them,—the animal directly poisoned alone perishes. and the other remains unharmed to the last."

Poisons vary as to their remote action, something like an elective affinity being manifested by certain articles for particular organs. The heart, for example, is specially affected by tobacco and the upas antiar; the pulmonary capillaries by tobacco and tartar emetic; the brain by narcotics generally; the spinal marrow by conia and nux vomica; various glands by mercury; and the alimentary canal by arsenic. It is further stated by writers on this subject that chromate of potash excites inflammation of the conjunctiva, and that ergot has been known to produce gangrene in different parts of the body, with dropping off of the toes.

The effects of poisons are modified by various circumstances, among which may be mentioned quantity, the state of aggregation, the chemical combination, mixture, difference of tissue, habit and idiosyncrasy, and diseased states of the body. Thus, arsenic in a large dose may prove speedily fatal, while in smaller quantities it may induce inflammation of the alimentary canal and a lingering death. White hellbore in minute quantities excites gastritis, and in larger doses brings on giddiness, convulsions and coma. The state of aggregation as a modifying circumstance is easily appreciated; poisons must be in solution, or at least be soluble, to exert their peculiar effects. The effect of chemical combination is seen in the case of all antidotes, in which poisons are rendered inert by their union with other bodies which themselves may be poisonous. The concentrated mineral acids and thecaustic alkalies are examples, both poisonous, but becoming harmless by combining. Mixture may increase the activity of a poison if thereby it is rendered more capable of reaching the circulation, but where the mixture is with the food in the stomach, as when taken during a meal, poisons are rendered less injurious by this circumstance. Arsenic is often ejected in such cases when it would be fatal on an empty stomach. Difference of tissue in the case of poisons which enter the circulation is a modifying circumstance, but where the poison exerts a corrosive influence the effect is nearly the same to what part soever applied. Carbonic acid is harmless in the stomach, but most deleterious in the lungs. The venom of the viper may also be swallowed with impunity, but if injected into a vein excites a train of alarming symptoms. Oxalic acid if not so concentrated as to produce irritation does not affect the stomach, and only becomes deleterious when it has passed from the digestive organs into the blood. Poisons which owe their power to absorption are most active when introduced into a recent wound; next to this in contact with the serous tissues; then in the mucous passages; and least of all when applied to the skin. Habit and idiosyncrasy are to be taken into the account, as many cases on record, and the observation of every one must make evident. Mercury is poisonous in the smallest doses to some constitutions; ipecacuan brings on a distressing asthma in some; tartar emetic is followed by alarming symptoms in others; while, as to the effect of habit, we see persons daily indulging in potations of ardent spirits, and others swallowing quantities of opium, which would be fatal to the uninitiated.

Our author makes an application of these facts to practice, thus:

"As an appendix to what has been said respecting the physiological action of poisons, and the causes by which it is liable to be modified, I shall here state shortly certain applications to the treatment of poisoning.

In the instance of internal poisoning, the great object of the physician is to administer an antidote or counter-poison. Antidotes are of two kinds. One kind takes away the deleterious qualities of the poison before it comes within its sphere of action, by altering its chemical nature. The other controls the poisonous action after it has begun, by exciting a contrary action in the system. In the early ages of medicine almost all antidotes were believed to be of the latter description, but in fact very few antidotes of the kind are known.

Chemical antidotes operate in several ways, according to the mode of action of the poison for which they are given. If the poison is a pure corrosive, such as a mineral acid, it will be sufficient that the antidote destroy its corrosive quality: Thus the addition of an alkali or earth will neutralize sulphuric acid, and destroy or at least prodigiously lessen its poisonous properties. In applying this rule care must be taken to choose an antidote which is either inert in itself, or if poisonous, is like the poison for which it is given, a pure corrosive or local irritant, and one whose properties are reciprocally neutralized.

If the poison, on the other hand, besides possessing a local action, likewise acts remotely through absorption, or by an impression on the inner coat of the vessels, mere neutralization of its chemical properties is not sufficient; for we have seen above that such poisons act throughout all their chemical combinations which are soluble. Here, therefore, it is necessary that the chemical antidote render the poison insoluble or nearly so; and insoluble not only in water, but likewise in the animal fluids, more particularly the juices of the stomach. The same quality is desirable even in the antidotes for the pure corrosives; for it often happens that in their soluble combinations these substances retain some irritating, though not any corrosive power. When we try by the foregoing criterions many of the antidotes which have been proposed for various poisons, they will be found defective; and precise experiments have in recent times actually proved them to be so.

The other kind of antidote operates not by altering the form of the poison, but by exciting in the system an action contrary to that established by the poison. On considering attentively, however, the phenomena of the action of individual poisons, it will be found exceedingly difficult to say what is the essence of a contrary action, and still more how that counteraction is to be brought about. Accordingly, few antidotes of the kind are known. Physiology or experience has not yet brought to light any mode of inducing an action counter to that caused by arsenic and most of the irritant class of poisons. It appears probable that the remote operation of lead may be sometimes corrected by mercury given to salivation, and that the violent salivation caused by mercury may be occasionally corrected by nauseating doses of antimony. But these are the only instances which occur to me at present of antidotes for irritant poisoning which operate by counteraction, unless we choose to designate by the name of antidote the conjunction of remedial means which constitute the antiphlogistic method of cure. In the class of narcotics we are acquainted with equally few constitutional antidotes, although the nature of the action of these poisons seems better to admit of them. Ammonia is to a certain extent an antidote for hydrocyanic acid, but by no means so powerful as some persons believe;and I am not sure that in this class of poisons we can with any propriety mention another antidote of the constitutional kind.

On the whole, then, it is chiefly among the changes induced by chemical affinities that the practitioner must look for counter-poisons; and the ingenuity of the toxicologists has thence supplied the materia medica with many of singular efficacy. When given in time, magnesia or chalk is an antidote for the mineral acids and oxalic acid, albumen for corrosive sublimate and verdigris, bark for tarter-emetic, common salt for lunar caustic, sulphate of soda or magnesia for sugar of lead and muriate of baryta, chloride of lime or soda for liver of sulphur, vinegar or oil for the fixed alkalis; and these substances act either by neutralizing the corrosive power of the poison, or by forming with it an insoluble compound.

"In recent times a new object in the treatment of poisoning has been pointed out by the discoveries made in its physiology. As it has been proved that many of the most deadly poisons enter the blood, and in all probability act by circulating with that fluid, so it has been inferred that an important object in the treatment is to promote their discharge by the natural secretions. In support of this reasonable inference it has been lately rendered probable by Orfila, as will be seen under the head of the treatment of the effects of arsenic, that it is of great advantage in some forms of poisoning to increase the discharge of urine.

"In the instance of external poisoning the main object of the treatment is to prevent the poison from entering the blood, or to remove it from the local vessels which it has entered.

"One mode, which has been known to the profession from early times, and after being long in disuse was lately revived by Sir D. Barry, and applied with success to man, is the application of cupping glasses to the part where the poison has been introduced. This method may act in various ways. It certainly prevents the farther absorption of the poison by suspending for a time the absorbing power of the vessels of the part covered by the cup. It also sucks the blood out of the wound, and may consequently wash the poison away with it. Possibly it likewise compresses the nerves around, and prevents the impression made by the poison on their sentient extremities from being transmitted along their filaments.

"Another mode is by the application of a ligature between the injured part and the trunk, so as to check the circulation. This is a very ancient practice in the case of poisoned wounds, and is known even to savages. But as usually practised it is only a temporary cure; as soon as the ligature is removed the effects of the poison begin. It may be employed, however, for many kinds of poisoning through wounds, so as to effect a radical cure. We have seen that most poisons of the organic kingdom are in no long time either thrown off by the system or decomposed in the blood. Hence if the quantity given has not been too large, recovery will take place. Now, by means of a ligature, which is removed for a short time at moderately distant intervals, a poison which has been introduced into a wound beyond the reach of extraction, may be gradually admitted into the system in successive quantities, each too small to cause death or serious mischief, and be thus in the end entirely removed and destroyed. Such is the practical application which may be made of some ingenious experiments performed not long ago by M. Bouillaud with strychnia, the poisonous principle of nux-vomica.

"The last mode to be mentioned is by the combination of the ligature with venesection, deduced by M. Verniere from his experimental researches formerly noticed. Suppose a fatal dose of extract of nux-vomica has been thrust into the paw of a dog; M. Verniere applies a tight ligature round the limb, next injects slowly as much warm water into the jugular vein as the animal can safely bear, and then slackens the ligature. The state of venous *plèthora* thus induced completely suspends absorption. The ligature is next tied so as to compress the veins without compressing the arteries of the limb, and a vein is opened between the wound and the ligature in such a situation, that the blood which flows out must previously pass through, or at least near the poisoned wound. When a moderate quantity has been withdrawn, the ligature may be removed with safety; and the extraction of the poison may be farther proved by the blood that has been drawn being injected into the veins of another animal; for rapid death by tetanus will be the result. It is not improbable that in this plan the preliminary production of venous plethora may be dispensed with; and then the treatment may be easily and safely applied to the human subject."

The evidence of general poisoning is examined by Dr. Christison with great ability. The circumstances to be attended to are, as to the symptoms, their suddenness and rapidity, their regularity of increase, uniformity, beginning soon after a meal, attacking the individual in a state of health; of which it may be said, that no single one is of itself conclusive, but that a number concurring a strong suspicion of poisoning might arise, especially if there were at the same time moral proof of the crime. The same circumstances, or the absence of them, in other cases may dispel any unfounded suspicions of poisoning. We give the following interesting case, cited by our author, as in point:

"The Crown Prince of Sweden while in the act of reviewing a body of troops on the 28th May, 1810, was observed suddenly to waver on his horse; and soon afterwards he fell off while at the gallop, was immediately found insensible by his staff, and expired in half an hour. As he was much beloved by the whole nation, a rumour arose that he had been poisoned; and the report took such firm root in the minds of all ranks, that a party of military, while escorting the body to Stockholm, were attacked near the city by the populace, and their commander, Marshal Fersen, murdered; and Dr. Rossi, the prince's physician, after narrowly escaping the same fate, was in the end obliged to quit his native country. Now, no other poison but one of the most active narcotics could have caused such symptoms, and none of them could have proved so quickly fatal unless given in a large dose. It was proved, however, that on the day of his death the prince had not taken any thing after he had breakfasted; and an interval of nearly four hours elapsed after that till he fell from his horse. This fact alone, independently of the marks of apoplexy found in the head after death, and the warning symptoms he had repeatedly had, was quite enough to show that he could not have died of poison, as it was incompatible with the known action of the only poisons which could cause the symptoms. This is very properly one of the arguments used by the Medical Faculty of Stockholm, which was consulted on the occasion."

In addition to these evidences we are to inquire into the morbid appearances found in the body of the deceased, and finally to draw all the information that can be afforded by chemical analysis. We must determine first what the poison is, and subsequently whether it was administered during the life of the individual, or inserted into some part of his body after death. It is to be searched for in the stomach, the texture of the liver, in the blood, the urine, or in the matter ejected from the stomach, or known to have been partaken of by the victim as food or drink.

Some years ago a steam boat was ascending the Ohio towards Pittsburgh, with passengers as usual from various parts of the country. The subject of abolition happened to be discussed, and Southern gentlemen spoke their opinions freely in the hearing of a black waiter or cook. That night shortly after supper, a number of the passengers were seized with nausea, vomiting, and other symptoms of poisoning, which in the case of one or two persons were so violent as to create much alarm; they, however, all recovered. On investigation it appeared that those only were affected who had taken corn bread at supper. A suspicion was hence excited that the black man, apprised of the fact that southern people are apt to eat corn bread, had mixed arsenic with it. He was charged with the crime but made his escape by jumping into the river. Some of the bread was sent to us for analysis, and was carefully examined in the Laboratory of the Medical Institute. Besides answering to all the tests for arsenic in the most conclusive manner, it gave curious collateral evidence of containing a poison in killing all the flies about the room, which were attracted to the water in which we boiled the bread.

Although poison may not be detected in the alimentary canal, or the organs, or in any of the articles of food, moral evidence of poisoning sometimes brought out, may establish the charge. Christison relates the case which follows as being of that character:

"A man of doubtful character and morals, well acquainted with chemistry and medical jurisprudence, and of disordered finances, was known to harbour a design on a friend's wife, who possessed a considerable fortune. At last he one morning invited the husband to breakfast with him at a tavern; and they breakfasted, in a private apartment, on beef-steaks, fried potatoes, eels, claret, and rum. They had scarcely commenced the meal when his guest complained of feeling unwell; and soon afterwards he vomited violently. This symptom continued, along with excruciating pain in the belly, for a long time before the prisoner sent for medical aid; indeed he did not procure a physician till the sufferer had been also attacked with very frequent involuntary purging. The physician, who, before seeing his patient, had received the prisoners explanation of the apparent cause of the illness, was led at first to impute the whole to cholera caught by exposure to cold; but on returning at seven in the evening and finding the gentleman had been dead for an hour, he at once exclaimed that he had been poisoned. On the body being inspected much external lividity was found, contraction of the fingers, and great inflammation, of the stomach and intestines, presenting an appearance like that of gangrene. On analyzing some fluid left in the stomach, no arsenic or other poison could be detected. The attention of the inspectors was turned specially to arsenic, because the prisoner was proved to have bought that poison, and to have made a solution of some white powder in his kitchen not long before the deceased died. The prisoner in his defence stated, that the deceased had been for some time much weakened by the use of mercury, and while in this state was seized with cholera; and he likewise attempted to make it probable that the man, in despair at his not recovering from a venereal disease, might have committed suicide. The council of physicians who were required to give their opinion on the case state on the contrary, that the deceased was a healthy man, without any apparent disposition to disease; that there was no pretext whatever for supposing suicide; that the inflammatory state of the stomach and bowels supplied strong probability of poisoning with arsenic, but not certain evidence; that acute gastritis from natural causes is always attended with constipation; that the deceased presented symptoms of stupor and other signs of derangement of the nervous system remarked in rapid cases of poisoning with arsenic; that cholera is very rare at the end of November, the season when this incident occurred; and that the poison might well be discharged by vomiting. Although all the prisoner's statements in defence were contradicted by satisfactory proof, and the medical evidence of poisoning was supported by a chain of the strongest general circumstances, the crime was considered by the court as not fully proved, because the prisoner could not be induced to confess, and because poison was not actually detected in the body. But on account of the very strong probability of his guilt, he was, in conformity with the strange practice of German courts in the like cases, condemned to fifteen years' imprisonment. In this instance—considering the kind of symptoms, their commencement during a meal, the rapidity of death, the signs of violent inflammation in the stomach after so short an illness, and the facility with which the absence of poison in the contents of the stomach may be accounted for, more especially if it be supposed that the poison was administered in solution,—I consider the medical evidence of death by poisoning so very strong, that, the general evidence being also extremely strong, the prisoner's guilt was fully demonstrated.

The moral evidence is of the greatest importance in questions of the sort. Thus, a woman was seized early in the morning with symptoms indicating that she had taken an acrid poison. A farmer, who had some knowledge of medicine, being called to see her, learned that she had breakfasted on porridge made by her husband of corn meal, and on analysis the meal proved to contain arsenic. Again; a woman was found who had evidently been poisoned with nitric acid; but whether she had taken it to commit suicide, or had received it from another was to be determined by circumstances. Stains were discovered on her face as if the acid had been spilled there in the act of forcing it down her throat, and on further examination similar stains were found on the hands of her husband, fixing the crime of murder upon him. In another case, a man was accused by a woman of attempting to poison her by putting copper into her soup. Chevalier examined the soup and detected an abundance of the poison in it; but not a trace of the copper could be discovered on the surface of the pot in which the soup had been boiled, or in the soup still remaining in it. It was consequently clear that the woman, who had some spite against the man, had herself introduced the poison.

(TO BE CONTINUED.)

